# Regression rate of high-grade cervical intraepithelial lesions in women younger than 25 years

**DOI:** 10.1007/s00404-022-06680-4

**Published:** 2022-07-21

**Authors:** Anne Ehret, Victoria Naomi Bark, Anne Mondal, Tanja Natascha Fehm, Monika Hampl

**Affiliations:** Department of Obstetrics and Gynecology, University Hospital of Duesseldorf, Duesseldorf, Germany

**Keywords:** CIN 3, Regression rate, Spontaneous regression, Conization

## Abstract

**Background/purpose:**

The incidence and clinical course of high-grade cervical intraepithelial lesions (CIN 2/3) are age dependent. In CIN 3, the recommended treatment is conization, which increases the risk of cervical insufficiency or premature deliveries. But data concerning spontaneous regression of CIN 3 are rare.

**Methods:**

Between 2007 and 2017, we identified 156 women under the age of 25 with CIN 2 (23%) or CIN 3 (77%), who had a consultation and were treated at the Colposcopy Unit, Hospital of Düsseldorf, Germany. This is a retrospective cohort study. These patients had colposcopical follow-ups every 4–6 months. Moreover, we analyzed various parameters to predict regression of cervical lesions in this age group.

**Results:**

Patients diagnosed with CIN 2 showed regression in 88% (*n* = 30) and women with CIN 3 had a regression rate of 29% (*n* = 34). Complete regression was observed in 86.7% of CIN 2 and 47.1% of CIN3. Mean time to regression was 21 M (months) [2–70 M]. 70.9% of the patients were treated by surgery (LEEP) after persistence or progression. We identified several predictors for regression of CIN 2/3 in young women: the regression rate of CIN2 is significantly higher than CIN 3 (*p* < 0.001). Clearance of HPV infections had significantly higher rates of regression compared to persisting HPV infections (*p* < 0.001). HPV-vaccinated women showed significantly higher regression rates (*p* = 0.009).

**Conclusions:**

These data show that an expectative close follow-up in women with CIN 3 younger than 25 is possible with regression rates of 29% also for CIN 3. Especially in women who were HPV vaccinated and those who cleared their HPV infection. A frequent colposcopical follow-up every 3–4 months is important for CIN 3 and every 6 months for CIN 2.

## What does this study add to the clinical work

**Table:** 

Young women, younger than 25 years, show spontaneous regression of CIN 3 with a regression rate of 29.1%. An observational management should be discussed with these patients, especially with HPV vaccinated women who showed more often spontaneous regression.	

## Introduction/background

Since introduction of cervical cancer screening in several, especially high-income, countries, the incidence and mortality of cervical cancer decreased markedly.

In Germany, cervical cancer screening was introduced in 1971, offering women yearly examinations of the cervix by cytology, or so-called “pap smear.” Within the last 15 years, the incidence and mortality of cervical cancer is largely stable [1]. The average age at initial diagnosis of invasive cervical cancer is 55 years [1].

The primary goal of secondary prevention of cervical cancer is to discover precancerous lesions of the cervix in order to prevent progression to invasive cancer.

The main cause of cervical cancer and its precursors is a persistent infection with human papilloma viruses (HPV) [2]. An asymptomatic infection is very common in young women, especially younger than 25 years, and in most cases, the HPV infection is recognized by the immune system and clears spontaneously without consequences [3]. The lifetime probability of HPV infection in sexual active adults is reported to be about 85–90% [3]. A persistent infection with high-risk HP viruses, especially the high-risk types 16 or 18, has a substantial risk to progress to cervical intraepithelial lesions within months to years, and to progress to cervical cancer within 7–15 years [4].

The course of HPV infection in squamous epithelium is well examined:

After sexual intercourse, women may acquire an HPV infection. Due to micro-lesions of the epithelium, the virus particles can “reach” the basal layer of the squamous epithelium and infect these basal cells [5]. This is called latent infection and can last for many years.

During permissive infection, the amplification cycle of the virus particle, including infectious descendants, takes place in epithelium cells [5]. These cells are called koilocytes. The viral reproduction cycle and the differentiation of the epithelium are aligned to each other.

In most cases, the human immune system is able to clear the HPV infection within 1–2 years [3].

About 10% of infected individuals do not clear the virus and develop persistent infection: HPV persists in the squamous cells and is replicated geared to the cell cycle of the epithelium [3].

In some cases, it could lead to a transforming infection: a turnover of normal to dysplastic or malignant cells. Risk factors are compromised immune system, HIV infection, smoking cigarettes and immunosuppressive medications [6].

The precancerous lesions of squamous epithelium, or so-called cervical intraepithelial lesions (CIN), can range from mild (CIN 1), moderate (CIN 2) to severe (CIN 3) lesions and carcinoma in situ. Adenocarcinoma in situ (AIS) is the glandular precursor for invasive endocervical adenocarcinoma.

International guidelines recommend a surgical treatment for high-grade intraepithelial lesions (CIN 2/3) and a watch-and-see-strategy for low-grade lesions (CIN1).

German guidelines suggest waiting up to 24 months in cases of CIN 1 by performing colposcopy every 6 months. CIN 2 can be monitored for at least 12 months as well. In case of persistence or progression to CIN 3, treatment by surgery is recommended [7].

The recommended treatment of histologically proven CIN 3 is surgery, only in young individuals a short-term observational period is possible [7].

The standard procedure is conization of the cervix uteri. Nowadays, a loop electrosurgical conization under colposcopic guidance is the gold standard (LEEP = loop electrosurgical excision procedure). Alternative procedure is laser conization. CIN 1–2 may also be treated by laser coagulation of the cervical lesion after biopsy for histology [7]. Conizations performed by cold knife are obsolete.

Specific complications are bleeding, cervical stenosis or shortened cervix during following pregnancies with the risk of premature rupture of membranes and preterm deliveries [8–10].

Especially in young women who have not decided their family planning, yet, surgical treatment should be indicated with restriction and caution.

As regression rates in mainly CIN 2 lesions are high, it is important to find special predictors, which can accurately predict which lesions may regress or not.

Table 1 summarizes some studies about observational management of CIN 2 in young women. The observed regression rates were between 39 and 71.1%, whereas the progression rates were about 8.3–16.6% [11–14].

**Table 1:** Studies about observational management of CIN 2 in young women, regression and progression rates during their observation period

Author, year	*n* (women with CIN 2 < 25 J/* < 21 J)	Regression rate	Progression rate
Fuchs K, 2007*	40	39%	8,3%
Munro A, 2016	924	59.5%	16.4%
Loopik DL, 2016	211	71.1%	16.6%
Moscicki AB, 2010	95	68%	15%

*Women with CIN2 < 21 years

The discrepancy in results may be explained by several factors:

In most studies focused on the regression rates, there was no differentiation between the CIN 2 and the CIN 3 group. Furthermore, p 16 immunohistochemistry was not performed. This can lead to misleading results in histology, as inflammation was misinterpreted as dysplasia in some cases.

In many studies, there were only a restricted number of patients, which makes it even more difficult to determine the numbers of regressions in an appropriate way.

Taken all together, regression rates and their appropriate predictors have to been analyzed in an accurate way to find out whether a conservative approach with short-term controls may be safe in young women and could help to avoid unnecessary surgery to preserve the cervix and reduce pregnancy complications.

## Patients and methods

This is a retrospective cohort study.

### Patient cohort

After ethical approval, we searched the in-house medical record database to identify patients younger than 25 years who had received a colposcopic and histologically verified diagnosis of CIN 2 or 3 between 2007 and 2017 at the Colposcopy Unit, Department of Obstetrics and Gynecology, University Hospital of Düsseldorf, Germany. We excluded histologically proven Adeno Ca in situ.

The patients had follow-ups with colposcopy, repeated Pap smear and HPV testing if indicated every 3–6 months to identify regression, persistence or progression of their cervical lesion. Moreover, we analyzed various parameters to predict regression of cervical lesions in this age group (< 25 years).

The following parameters were documented and analyzed from each patient if available:

- Age at the time of initial diagnosis
- Cervical cytology result (PAP smear result)
- Date of first and the following biopsies and histologically result of each biopsy
- HPV status and classification as low-risk or high-risk types, including HPV typing
- Immunohistochemistry with KI67 and p16INK4a biomarkers
- HPV vaccination (before or after first intercourse?) and history of HPV infections
- BMI
- Smoker/non-smoker
- Family history of cancers
- Immunodeficiency
- Genital co-infection and therapy
- Current medications, including birth control pills
- Gynecological history, including recent pregnancies, births, methods of contraception
- History of gynecological surgery

If a surgical treatment of CIN was needed, the following parameters were collected:

- Date of surgery
- Histologically report of grade of CIN, size, and margins
- The time of surgery distributed in direct vs. secondary direct: surgery within 3 months after initial diagnosis vs. secondary: an extended control interval with consecutive surgery in case of missing regression.

The follow-ups were scheduled every 3–4 months for CIN 3 and every 6 months for CIN 2.

All cytology, HPV testing, and histological examinations as well as immunohistochemistry had been performed at the local institute of Cytology and Pathology, University Hospital of Düsseldorf, Germany.

### Statistical analysis

All statistical analyses were performed using the program SPSS Statistics 25 (IBM Corp., Armonk, NY, USA). Categorical data were compared using the Pearson *χ2* test, while continuous data were compared using the student *t*-test. *P* < 0.05 was considered statistically significant.

Binary logistic regression analysis was used to analyze different predictors for regression.

### Ethical approval and consent to participate

Ethical approval was obtained from the ethics committee at the University Hospital Düsseldorf, Germany. All patients were informed about the treatment/follow-up options and written consent was obtained from all the women.

## Results

### Patient cohort

Between 2007 and 2017, we identified 156 women younger than 25 with histologically confirmed CIN 2 or CIN 3 who had a consultation and were treated at the Colposcopy Unit, Department of Obstetrics and Gynecology, University Hospital of Düsseldorf, Germany.

For data analyses, five patients had to be excluded because of missing follow-ups.

At the first visit, colposcopy with photo documentation and cervical tissue biopsy was performed to confirm high-grade lesion (CIN 2 or 3), including pap smear and HPV testing if not a recent result was available from the referring outpatient clinic.

During the close follow-up visits, colposcopy and cytology were repeated. If regression or progressive disease was suspected in comparison to the preceding/the initial diagnosis, cervical biopsy was repeated. If indicated HPV testing was repeated after an at least 6-month time interval.

Regression of CIN was diagnosed by colposcopy and confirmed by either cervical biopsy or by analyzing the cone specimen, if a LEEP was performed.

The median age was 21 years (range 15–25 years, *n* = 151). In total, 34 patients (22.5%) had the diagnosis of CIN 2 by performing a biopsy of the cervix under colposcopic guidance, whereas 77.5% (*n* = 117) had a histologically proven CIN 3. In 19 cases, the histology was documented as “CIN 2 to CIN 3”, so these cases were analyzed in the group of CIN 3.

### HPV status/testing

HPV testing was performed using the Digene Hybrid capture test system (Qiagen) or the Cobas HPV Test (Roche). In total, 99.3% of the women were tested HPV DNA positive. HPV infection with one of the high-risk HPV type was found in 97 patients (64.2%) and 28 patients (18.5%) had multiple infection with several low-risk and high-risk HPV types, 4 patients (2.6%) were infected by a low-risk type only, and in 13.9% (*n* = 21), there was just recorded “HPV DNA positive” without specification of low-or high-risk type. One patient (0.7%) was tested HPV DNA negative.

### Indication for secondary surgery

If there was no regression of CIN 3, or long-term persistence or progression of CIN 2, secondary surgical treatment by LEEP was indicated.

The time between diagnosis of cervical dysplasia and consecutive surgery by LEEP was different case by case and is part of the following analysis (see below).

We distinguish between complete regression to normal cervical tissue or partial regression to CIN 1 or CIN 2 (in CIN 3, respectively).

70.9% of the patients (*n* = 107) underwent surgery. Indication of surgical treatment was a persistent CIN 3 in 101 cases (94.4%), and 6 patients (5.6%) had a persisting CIN 2.

In these six cases of CIN 2, there was no regression for 25.85 months in average (range: 2.6–26 months), so the LEEP was indicated/or strong patients’ wish. None of them had progression to CIN 3.

The final histopathological diagnosis of the cone specimen showed a regression in two of the six patients with preoperatively persisting CIN 2 for several years (3.4 years and 5.8 years).

### Time period between diagnosis and surgical treatment

A quarter of the patients with CIN 3 (27.1%, *n* = 29) and one patient with CIN 2 underwent surgery without “wait and see” within three months after diagnosis (wish of patient, refusal to wait). The majority of LEEPs due to CIN 3 (72.9%, *n* = 78) were performed after a period of expectative strategy.

**Fig. 1:**
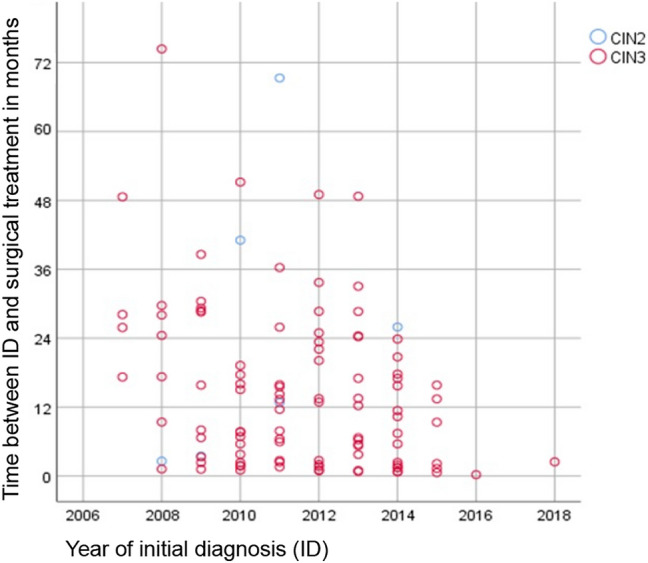
Time periods between diagnosis of CIN 2 or 3 until date of surgical treatment between 2007 and 2018 (*n* = 107)

The time lapse for “wait and see” until surgical treatment ranges for CIN 2 from 3.48 to 69.32 months; (average 30.5 months) and for CIN 3 from 0.23 to 74.38 months (average 14.52 months), see Fig. 1.

### Progression or regression during “wait and see “?

In total, we observed regression of dysplasia in 42.4% of the patients (*n* = 64).

Almost a third of these patients younger than 25 years (27.8%, *n* = 42) showed a complete regression of CIN 2 or CIN 3.

**Fig. 2:**
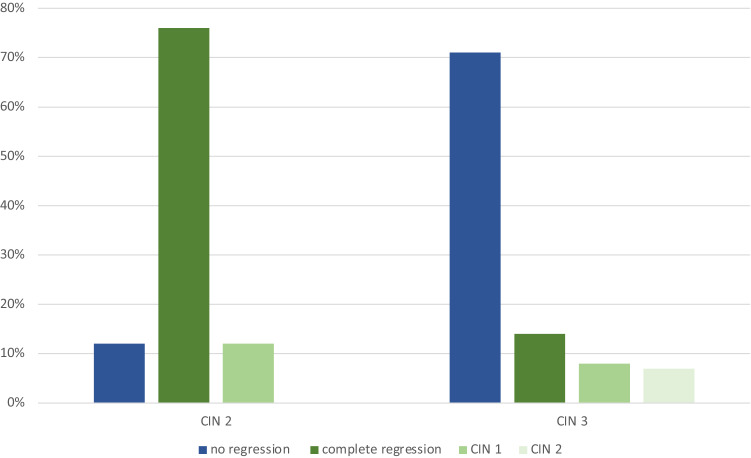
Diagnoses at the end of “wait and see” period/in postoperative specimen for initial diagnosis of CIN 2 (*n* = 34) and CIN 3 (*n* = 117)

Patients with CIN 2 had in 12% (*n* = 4) a persisting disease with no regression and no progression (Fig. 2). Therefore, in 88% (*n* = 30) of the cases, we observed a regression in severity of dysplasia: four patients (12%) had a partial regression to CIN 1 and 76% (*n* = 26) had a complete regression during the “wait and see” period.

Patients with high-grade dysplasia, CIN 3, had in 71% (*n* = 83) persisting disease within the follow-up period without progression or regression (Fig. 2). There was no patient developing invasive disease in the follow-up period. A complete regression of the CIN 3 to normal cervical tissue was diagnosed in 16 cases (14%), a partial regression to CIN 1 in 10 cases (9%) and to CIN 2 in 8 cases (7%), see Fig. 2. Therefore, the overall regression rate in CIN 3 was 29.1% (34/117).

In total in our study, patients with initial diagnosis of CIN 2 showed partial or complete regression of cervical dysplasia in 88% (*n* = 30). Patients with CIN 3 had a partial or complete regression rate of 29.1% (*n* = 34).

Taken together, the rate of complete spontaneous regression of CIN 2/3 in women younger than 25 years was (27.8%, *n* = 42).

### Time period between initial diagnosis and regression (partial and complete)

The time period between initial diagnosis of CIN 2/3 and the diagnosis of regression of the cervical dysplasia varied between < 3 months and up to more than 5 years.

**Fig. 3:**
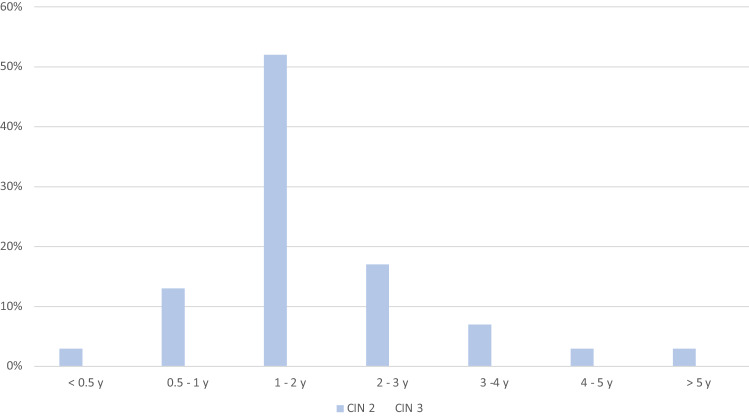
Time period between initial diagnosis and regression (partial and complete) for CIN 2 (*n* = 34) and CIN 3 (*n* = 117)

But, the majority of the patients who show regression (partial and complete) in dysplasia stage, we diagnosed the changes within the first 2 years: 70% (*n* = 21) of CIN 2 cases and 68% (*n* = 23) of CIN 3 cases (Fig. 3). After 3 years, 5 more patients of CIN 2 (16.7%) and 6 more of CIN 3 (17.6%) showed improved or normal results of cervical examination (pap, biopsy, colposcopy, HPV DNA testing).

Thus, regarding all patients with diagnosis of CIN 2, we recognized a regression of dysplasia in 21/34 cases (61.7%) within 2 years. The spontaneous regression of CIN3 was lower: within 2 years, 23/117 cases (19.6%) showed improvement of their cervical dysplasia and 29/117 (24.8%) within 3 years.

### Prediction of regression of CIN 2 and CIN 3

#### HPV vaccination

**Fig. 4:**
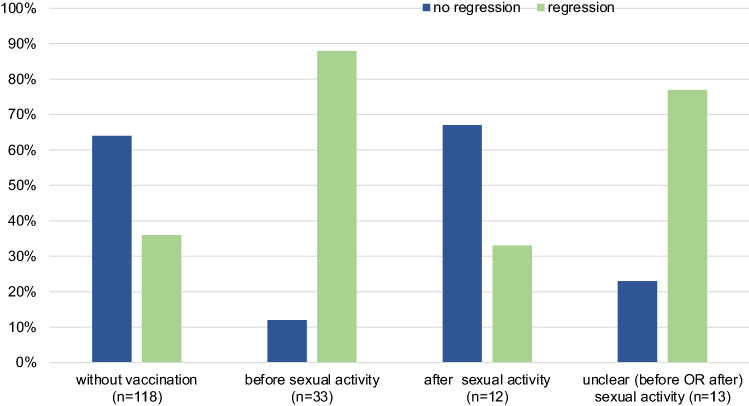
Regression of CIN 2 and 3 depending on time of HPV vaccination (*n* = 151)

33 of 151 patients had prior HPV vaccination. Patients without a vaccination (*n* = 118) showed in 36.4% a regression (*n* = 43) and no regression in 63.6% (*n* = 75), see Fig. 4.

The patients who got vaccinated against HPV before first sexual intercourse (*n* = 8) had the best results: 87.5% (*n* = 7) had regressive results (Fig. 4).

Meanwhile, only 4 of 12 patients (33%) who got vaccinated after the first sexual intercourse had a spontaneous regression of their high-grade lesion (CIN 2 or 3). For 13 patients, the timeline of HPV vaccination and first sexual contact was unclear or not well documented. But, 76.9% (*n* = 10) of these HPV-vaccinated patients also showed a regression.

In conclusion, after HPV vaccination at any time, the chance of regression of CIN 2 or 3 is significantly higher compared to the patients without HPV vaccination (*p* = 0.009).

#### Clearance of HPV infection

The clearance of HPV high-risk infection significantly predicts the regression of severe cervical dysplasia, *p* < 0.001. Patients with HPV clearance (*n* = 34) showed a regression of cervical dysplasia in 79.4% of the cases (*n* = 24). Those patients without HPV clearance during observation time (*n* = 113) developed regressive cervical disease in only 29.2% (*n* = 33).

#### Initial diagnosis/histology and regressive disease

As described above, the regression rate for initial diagnosis of CIN 2 (88.2%, *n* = 30) was significantly higher than in patients with CIN 3 (29.1%, *n* = 34; *p* = 0.0002).

Therefore, the diagnosis of CIN 2 has a significantly higher prediction of developing a regression of cervical dysplasia compared to initial diagnosis of CIN 3 (*p* < 0.001, OR = 0.06 [95% KI: 0.02; 0.17]).

#### Smoking

In this cohort, 75 patients were cigarette smokers and 76 non-smokers. A spontaneous regression of CIN 2 or 3 was seen in 38.7% (*n* = 29) of the smoker and 46.1% (*n* = 35) of non-smoker. In our study of young women (< 25 years of age), smoking is not a predictor of regression of CIN 2 or 3, *p* = 0.359, OR = 0.74 [95% KI: 0.38; 1.41].

#### Contraception

There was no significant correlation between the hormonal contraception and the prediction of CIN 2/3 regression, *p* = 0.483, OR = 0.78 [95% KI: 0.39; 1.55].

#### BMI (body mass index)

There was no significant correlation between BMI of the patients and regression of cervical dysplasia (*p* = 0.761). In this cohort, the regression rate of normal weight patients (BMI 18.5–24.9 kg/qm) was 40.2% (*n* = 47), of underweight (BMI < 18.5 kg/qm) 52.9% (*n* = 17), of overweight (BMI = 25–29.9 kg/qm) 46.2% (*n* = 13) and of patients with adiposity (BMI ≥ 30 kg/qm) 33.3% (*n* = 3).

## Discussion

Nowadays, most of the women younger 25 years have not decided their family planning, yet. Therefore, uterine surgery such as loop conizations should be recommended restrictively and cautiously. Thus, “watch and wait” strategies for cervical dysplasia especially in young women need to be discussed.

So far, there are only a few data available about regression rates, progression rates and times of observation periods for CIN 3 in the young age group, in contrast to several studies concerning regression rates in CIN 2.

The regression rates in CIN 2 lesions in previous studies vary from 39 to 71% (see Table 1), whereas in CIN 3 lesions, the rates vary from 1.3 to 38% [11–18].

A large Australian retrospective cohort study published by Munro et al. 2016 analyzed conservative management of 924 Western Australian women aged 18–24 years diagnosed with CIN2 on cervical biopsy [12]. In their cohort, the 2-year regression rate for CIN2 was 59.5% and the progression rate was 16.4% (CIN 3 and AIS). None of the women progressed to invasive cancer.

In the same year, Loopik et al. published another retrospective cohort study (*n* = 211) of women younger than 25 years with CIN2 managed conservatively [13]. The regression rate was even better with 71.1%) showed regression, and 16.6% of the young women progressed (no cancer), with a median follow-up of 15.1 months. Smoking was a risk factor for progression (hazard ratio 2.40, *p* = 0.006).

A prospective study by Moscicki et al. examined the progression and regression including risk factors of young women (13–24 years) and CIN 2, *n* = 95 [14]. They found a 2-year regression rate of 63% and 68% after 3-year follow-up. The progression to CIN 3 was 15% and similar to the above studies. Non-progression and regression were associated with non-persistence of HPV infection (hazard ratio 0.40; 95% CI 0.22–0.72) or oral contraceptive use (hazard ratio 0.85; 95% CI 0.75–0.97).

Another small cohort study evaluated regression rates among adolescents (aged < or = 21) with CIN2 and managed expectantly [11]. 36 young women could be followed conservatively and regression after a median follow-up time of 378 days was documented in 14 (39%). The authors defined regression as complete regression to normal cytology and biopsy/colposcopy. 19 patients had a CIN 1 or mildly abnormal cytology results and only 3 patients developed a persistence or progression. Therefore, in total, 92% of women younger than 21 and CIN 2 had regressive results during conservative management.

In our cohort study of 151 cases, the regression rate of CIN 2 was 88% (*n* = 30) and even 76% (*n* = 26) for complete regression, which is higher than the published data so far, and without any case of progression during the observation period of up to 5 years. Reasons for these might be a very high standard and experience of the examiners at our colposcopy unit and good education and reassurance to the patients that the expectative management is safe, and they have a good chance to clear their HPV infection/lesion.

For CIN 3, little conclusive data are available—the published regression rates vary between 1.3 and 38% [15–18]. In our cohort, 29% of the patients younger than 25 years (*n* = 34) developed regressive results after an observation period of up 5 years, and even 14% (*n* = 16) had complete regression of cervical dysplasia. We encountered no case of progression and of invasive disease during observation time.

Motamedi et al. published 2015 a retrospective analysis of 635 cases with CIN 3. Their regression rate of CIN 3 was 1.3% (*n* = 8). But, they detected 12 invasive carcinomas after conization in the cone tissue. Therefore, the rate of undetected cervical cancer was higher than the regression rate and they concluded that observational management of CIN 3 is not justifiable [15]. It should be noted that in this study, the median women’s age was 32 years, and there is no age-related analysis concerning regression rate in the younger age group available.

Moreover, many studies did not distinguish between CIN 2 and CIN 3. Often, they did not use immunohistochemistry for histological diagnoses, which might cause mistakes in classification. We must remark, in our cohort also 19 cases could not be distinguished between CIN 2 or 3, so we treated them as CIN 3. One cannot rule out the possibility that the patients “just” had a CIN 2, so the rates might be biased.

Munk et al. documented a regression rate of CIN 2–3 from 5% after 9 weeks observation up to 38% for a longer period but had a small sample size of 61 patients in total [16].

The aim of the study was to evaluate the hypothesis that “curative” punch biopsies are the reason for CIN 3 regression. With a rising regression rate by longer observation periods and regression despite positive resection margins of the cervical biopsies, they could disprove this hypothesis.

A meta-analysis done by Zhang et al. found regression rates for CIN 2 of 50.85% and of 36.31% for a group of CIN 2/3 (without differentiation of those) [17]. The median age was 28.23 years. Therefore, the trend of these results matches with our regression rates and confirm our data quite nicely.

Nonetheless, our results also show that the chance of regression of CIN 2 is much higher than CIN 3 (*p* < 0.001). CIN2 are less aggressive and rarely end in cervical cancer, so it has a much higher chance to regress spontaneously [18]. In our hands, young women with CIN 2 never get the indication for surgery right away but always are treated observationally, to the point that in our hands, immediate resection of CIN 2 in young women without risk factors is considered unnecessary surgical treatment. Based on the results of this study in our institution, also CIN 3 can be managed observationally in young women without any risk factors and informed consent, because the rate of regression in a third of those women is quite high and justifies the “wait and control” management. The reliability of the women and short-term follow-up examinations are precondition for this kind of management.

Looking at the time interval for regression, the retrospective cohort study published by Munro et al. 2016, (*n* = 924) evaluated a regression rate of CIN 2 of 59.5% within 2 years. These results perfectly match with our findings: we exploited a regression of CIN 2 in 61.7% (*n* = 21) of our cases within 2 years [12].

Lee et al. 2018 also analyzed the outcomes of conservative management of CIN 2 and 3 in young women and found regression rate (partial and complete) of CIN 2 of 74.7% (*n* = 74), which is a just a bit lower than the regression rate of 88% in our cohort [19]. The median time to regression was 10.8 months. In this study, the regression rate for CIN 3 was 21.6% (*n* = 11), but the median time to regression was not reached. In our study, the spontaneous regression of CIN3 was quite similar with 19.6% within 2 years (*n* = 23) and 24.8% within 3 years (*n* = 29).

To summarize, for young patients with CIN3 without any risk factors, an observation period should be recommended with informed consent of the patient. But for this conservative management, mechanisms are necessary to ensure that these women return to their follow-up, at least every 3–6 months.

Due to small sample sizes of published data to this point, larger studies are still required.

### HPV vaccination

We recognized that HPV vaccination at any time increases the chance of regression of CIN 2 or 3 significantly compared to the patients without any HPV vaccination (*p* = 0.009, Fig. 4).

The most effective time point of HPV vaccination regarding regression of cervical dysplasia was before initiation of sexual intercourse [(regression rate of 87.5% (*n* = 7)].

This is just one reason to recommend HPV vaccination at the age of 9–14 years to girls and boys as per many guidelines, e.g., the German guideline does [7]. The viral protection is up to 100% if the girls got vaccinated before sexual intercourse [20].

Different studies demonstrated the high effectiveness of the HPV vaccine in very young women regarding prevention of CIN 2 + [20, 21].

But even after first sexual contact, the HPV vaccination is effective [22]. HPV infections can also appear to be the result of new exposure or reactivation of latent HPV infection that was previously undetectable [23].

Since the current HPV vaccine protects against 9 different high-risk HPV types and HPV 6/11 and it is very unlikely to get exposed to multiple HPV types at once, the vaccination will achieve a broader protection anyway [24].

Based on these data, the FDA extended the recommendation of HPV vaccination for any women younger than 45 years [25].

Moreover, several studies could show that HPV vaccination after conization is also effective to reduce the recurrence of CIN 2 + [26–31] .

### Contraception

The role of oral hormonal contraception in developing HPV-related cervical dysplasia is controversial. We did not find any correlation between oral contraception and the prediction of CIN 2/3.

Syrjanen et al. as well as Longatto-Filho et al. showed that oral contraceptives are not an independent risk factor for CIN or HPV infections [32, 33].

On the contrary, Oh HY et al. did find evidence that development of CIN 2/3 is higher in women taking oral contraceptives (independent of the duration of intake) [34].

A metanalysis published by Asthana et al. 2020 also concluded higher risk of invasive cervical cancer (Adenocarcinoma OR of 1.77 (95% CI 1.4, 2.24), squamous cell carcinoma 1.29 (95% CI 1.18, 1.42) and carcinoma in situ 1.7 (95% CI 1.18, 2.44)) [35].

Moreover, concerning invasive cervical cancer, combined oral contraceptives are classified by the International Agency for Research on Cancer as a cause of cervical cancer [36]. They analyzed 24 epidemiological studies that showed an increased relative risk for long-term combined oral contraceptives users for cervical cancer.

However, the correlation between oral hormonal contraception and development of HPV-related dysplasia is still controversial and complex to answer. Prospective studies were desirable to answer this question in detail.

## Conclusion

In total, the regression rate (partial and complete) for CIN 2 was 88.2% (*n* = 30) and significantly higher than the regression rate (partial and complete) for CIN 3 with 29.1% (*n* = 34), (*p* = 0.0002).

Within 2 years of a “wait and see” approach, 70% (*n* = 21) of CIN 2 and 68% (*n* = 23) of CIN 3 showed either a partial or even complete regression. None of the patients in this cohort (*n* = 151) developed progression of cervical dysplasia or even progressed to cervical cancer.

This allows us to prolong the observation period and the follow-up interval in patients with initial diagnosis of CIN 2 younger than 25 years, in order to reduce the necessity of surgical treatment of the uterine cervix. We suggest a follow-up including colposcopy ± biopsy to be repeated every 6 months for 2 years.

Moreover, we should check the indication for immediate surgical treatment in women with initial diagnosis of CIN 3 without any risk factors and being younger than 25 years. The observational strategy is justified for at least one year under close supervision by an expert team with colposcopy + biopsy/HPV testing if needed every 3–4 months.

To predict the regression of CIN 2 and 3 in patients younger than 25 years while “wait and see”, we found three significant factors: the initial histological diagnosis (CIN 2 or 3), the clearance of HPV infection and prior HPV vaccination.

We should also add the repetition of HPV high-risk testing at the follow-up examinations to determine potential HPV clearance.

Larger prospective cohort studies or case–control studies to examine the spontaneous regression rates of severe cervical dysplasia are needed, including data about progression rates. Furthermore, increasing vaccination rates of HPV might influence the regression rates. These data are important for comprehensive patient information.

## Data Availability

Data cannot be shared publicly according to data protection act of German regulation.

## References

[1] Robert Koch Institute (2021) Cancer in Germany 2017/2018, 3.19 cervical cancer 2017, pp 86–89

[2] IARC Working Group on the Evaluation of Carcinogenic Risks to Humans (2012) Biological agents. A review of human carcinogens. IARC Monogr Eval Carcinog Risks Hum 100(Pt B):1–441 PMC4781184 23189750

[3] Schiffman M, Castle PE, Jeronimo J, Rodriguez AC, Wacholder S (2007). Human papillomavirus and cervical cancer. Lancet.

[4] Stuebs FA, Gass P, Dietl AK (2021). Human papilloma virus genotype distribution in women with premalignant or malignant lesions of the uterine cervix. Arch Gynecol Obstet.

[5] Doorbar J, Quint W, Banks L (2012). The biology and life-cycle of human papillomaviruses. Vaccine.

[6] Grulich AE, van Leeuwen MT, Falster MO, Vajdic CM (2007). Incidence of cancers in people with HIV/AIDS compared with immunosuppressed transplant recipients: a meta-analysis. Lancet.

[7] Hillemanns P, Friese K, Dannecker C, Klug S, Seifert U, Iftner T, Hädicke J, Löning T, Horn L, Schmidt D, Ikenberg H, Steiner M, Freitag U, Siebert U, Sroczynski G, Sauerbrei W, Beckmann MW, Gebhardt M, Friedrich M, Münstedt K, Schneider A, Kaufmann A, Petry KU, Schäfer APA, Pawlita M, Weis J, Mehnert A, Fehr M, Grimm C, Reich O, Arbyn M, Kleijnen J, Wesselmann S, Nothacker M, Follmann M, Langer T, Jentschke M (2019). Prevention of Cervical Cancer: Guideline of the DGGG and the DKG (S3 Level, AWMF Register Number 015/027OL, December 2017)—Part 2 on triage treatment and follow-up. Geburtshilfe Frauenheilkd.

[8] Nitahara K, Fujita Y, Tanaka D (2021). Laser vaporization of the cervix is associated with an increased risk of preterm birth and rapid labor progression in subsequent pregnancies. Arch Gynecol Obstet.

[9] Kyrgiou M, Koliopoulos G, Martin-Hirsch P (2006). Obstetric outcomes after conservative treatment for intraepithelial or early invasive cervical lesions: systematic review and meta-analysis. Lancet.

[10] Bruinsma FJ, Quinn MA (2011). The risk of preterm birth following treatment for precancerous changes in the cervix: a systematic review and meta-analysis. BJOG.

[11] Fuchs K, Weitzen S, Wu L, Phipps MG, Boardman LA (2007). Management of cervical intraepithelial neoplasia 2 in adolescent and young women. J Pediatr Adolesc Gynecol.

[12] Munro A (2016). Spontaneous regression of CIN2 in women aged 18–24 years: a retrospective study of a state-wide population in Western Australia. Acta Obstet Gynecol Scand.

[13] Loopik DL, Doucette S, Bekkers RLM (2016). Regression and progression predictors of CIN2 in women younger than 25 years. J Low Genit Tract Dis.

[14] Moscicki AB, Ma Y, Wibbelsman C (2010). Rate of and risks for regression of cervical intraepithelial neoplasia 2 in adolescents and young women. Obstet Gynecol.

[15] Motamedi M, Böhmer G, Neumann HH, von Wasielewski R (2015). CIN III lesions and regression: retrospective analysis of 635 cases. BMC Infect Dis.

[16] Munk AC (2007). Cervical intraepithelial neoplasia grade 3 lesions can regress. APMIS.

[17] Zhang J, Lu CX (2019). Spontaneous regression of cervical intraepithelial neoplasia 2: a meta-analysis. Gynecol Obstet Invest.

[18] Mark K (2019). Rates of regression of cervical dysplasia between initial biopsy and excisional procedure in routine clinical practice. Arch Gynecol Obstet.

[19] Lee MH, Finlayson SJ, Gukova K (2018). Outcomes of conservative management of high grade squamous intraepithelial lesions in young women. J Low Genit Tract Dis.

[20] FUTURE II Study Group (2007). Prophylactic efficacy of a quadrivalent human papillomavirus (HPV) vaccine in women with virological evidence of HPV infection. J Infect Dis.

[21] Garland SM, Hernandez-Avila M, Wheeler CM (2007). Quadrivalent vaccine against human papillomavirus to prevent anogenital diseases. N Engl J Med.

[22] Castellsagué X, Muñoz N, Pitisuttithum P, Ferris D, Monsonego J, Ault K, Luna J, Myers E, Mallary S, Bautista OM, Bryan J, Vuocolo S, Haupt RM, Saah A (2011). End-of-study safety, immunogenicity, and efficacy of quadrivalent HPV (types 6, 11, 16, 18) recombinant vaccine in adult women 24–45 years of age. Br J Cancer.

[23] Winer RL, Hughes JP, Feng Q, Stern JE, Xi LF, Koutsky LA (2016). Incident detection of high-risk human papillomavirus infections in a cohort of high-risk women aged 25–65 years. J Infect Dis.

[24] Harper DM, Franco EL, Wheeler CM (2006). Sustained efficacy up to 4 5 years of a bivalent L1 virus-like particle vaccine against human papillomavirus types 16 and 18: follow-up from a randomised control trial. Lancet.

[25] Food and Drug Administration Oct 5 2018 Summary Basis of Regulatory Action—GARDASIL 9Available at: https://www.fda.gov/media/117054/download uploaded Oct 20th 2021

[26] Joura EA, Garland SM, Paavonen J, Ferris DG, Perez G, Ault KA, Huh WK, Sings HL, James MK, Haupt RM (2012). Effect of the human papillomavirus (HPV) quadrivalent vaccine in a subgroup of women with cervical and vulvar disease: retrospective pooled analysis of trial data. BMJ (Clinical research ed).

[27] Garland SM, Paavonen J, Jaisamrarn U (2016). Prior human papillomavirus-16/18 AS04-adjuvanted vaccination prevents recurrent high grade cervical intraepithelial neoplasia after definitive surgical therapy: Post-hoc analysis from a randomized controlled trial. Int JvCancer.

[28] Kang WD, Choi HS, Kim SM (2013). Is vaccination with quadrivalent HPV vaccine after loop electrosurgical excision procedure effective in preventing recurrence in patients with high-grade cervical intraepithelial neoplasia (CIN2-3)?. Gynecol Oncol.

[29] Ghelardi A, Parazzini F, Martella F, Pieralli A, Bay P, Tonetti A, Svelato A, Bertacca G, Lombardi S, Joura EA (2018). SPERANZA project: HPV vaccination after treatment for CIN2. Gynecol Oncol.

[30] Jentschke M, Kampers J, Becker J, Sibbertsen P, Hillemanns P (2020). Prophylactic HPV vaccination after conization: a systematic review and meta-analysis. Vaccine.

[31] Zang L, Hu Y (2021). Risk factors associated with HPV persistence after conization in high-grade squamous intraepithelial lesion. Arch Gynecol Obstet.

[32] Syrjanen K (2006). Oral contraceptives are not an independent risk factor for cervical intraepithelial neoplasia or high-risk human papillomavirus infections. Anticancer Res.

[33] Longatto-Filho A (2011). Hormonal contraceptives and the length of their use are not independent risk factors for high-risk HPV infections or high-grade CIN. Gynecol Obstet Invest.

[34] Oh HY (2016). Association of combined tobacco smoking and oral contraceptive use with cervical intraepithelial neoplasia 2 or 3 in Korean women. J Epidemiol.

[35] Asthana S, Busa V, Labani S (2020). Oral contraceptives use and risk of cervical cancer-a systematic review & meta-analysis. Eur J Obstet Gynecol Reprod Biol.

[36] Appleby P, Beral V, Berrington de González A, Colin D, Franceschi S, Goodhill A, Green J, Peto J, Plummer M, Sweetland S, International Collaboration of Epidemiological Studies of Cervical Cancer (2007). Cervical cancer and hormonal contraceptives: collaborative reanalysis of individual data for 16,573 women with cervical cancer and 35,509 women without cervical cancer from 24 epidemiological studies. Lancet.

